# O.4.2-5 An eight-week pa intervention improves FMS & self efficacy in children with underlying conditions

**DOI:** 10.1093/eurpub/ckad133.180

**Published:** 2023-09-11

**Authors:** Eoin Andrew Kaar, Tara Coppinger, Mai O'Leary

**Affiliations:** Munster Technological University, Ireland; eoin.kaar@mycit.ie; Munster Technological University, Ireland; eoin.kaar@mycit.ie; Munster Technological University, Ireland; eoin.kaar@mycit.ie

## Abstract

**Purpose:**

To evaluate the effectiveness of the “FunFit” pilot intervention, designed to support children awaiting a therapy service within the Irish health system to increase their physical activity (PA) levels and improve fundamental movement skills (FMS). FunFit aims to provide children with a positive experience of PA in a non-competitive, community-based environment. This is a novel collaborative intervention approach for children with underlying conditions in Ireland.

**Methods:**

A total of 14 children (8 male, 6 female) aged 8-12 years (mean age: 9.19 ± 1.03) participated in the eight-week intervention. Trained exercise professionals delivered 1-hour PA classes each week. Pre and post intervention, a PA questionnaire assessed self-efficacy, enjoyment of PA and days of PA per week. 12 FMS (6 locomotor & 6 object control) skills were assessed using the Test of Gross Motor Development – 2 (TGMD-2). Paired T-Tests and Wilcoxon Signed Rank Tests were conducted to analyse improvements.

**Results:**

Nearly all (91.67%) children (N = 13) did not meet the recommended levels of PA before, or after, the intervention and PA levels remained low post intervention (mean days of PA per week: 4.33 ± 1.55). Yet, improvements in overall total locomotor (P = 0.012, effect size (ES) = 0.589), total object control (P = 0.034, ES = 0.499) and total FMS (P = 0.008, ES = 0.629), were evident after the intervention. A positive improvement in enjoyment of PA (P = 0.014, ES = 0.502), and in self efficacy (P = 0.035, ES = 0.430), were also reported after the intervention.

**Conclusions:**

An eight-week PA intervention for children with underlying conditions did not result in children reaching recommended levels of PA. Yet, improvements in FMS, self-efficacy and enjoyment of PA were evident. This evidence suggests that a focus away from PA levels in interventions is needed with these individuals; and instead, providing an intervention that promotes enjoyment of PA in a non-competitive environment in the community setting could be more effective. By doing so, children could be supported in developing their FMS, which may then have a positive knock-on effect on their PA levels over a longer time period.

